# Effects of the Amino Acid Constituents of Microcystin Variants on Cytotoxicity to Primary Cultured Rat Hepatocytes

**DOI:** 10.3390/toxins6010168

**Published:** 2013-12-30

**Authors:** Kumiko Shimizu, Tomoharu Sano, Reiji Kubota, Norihiro Kobayashi, Maiko Tahara, Tomoko Obama, Naoki Sugimoto, Tetsuji Nishimura, Yoshiaki Ikarashi

**Affiliations:** 1 Division of Environmental Chemistry, National Institute of Health Sciences, 1-18-1 Kamiyoga, Setagaya-ku, Tokyo 158-8501, Japan; E-Mails: reijik@nihs.go.jp (R.K.); norihiro.kobayashi@nihs.go.jp (N.K.); tahara@nihs.go.jp (M.T.); obama@nihs.go.jp (T.O.); ikarashi@nihs.go.jp (Y.I.); 2 Center for Environmental Measurement and Analysis, National Institute for Environmental Studies, 16-2 Onogawa, Tsukuba-shi, Ibaraki 305-8506, Japan; E-Mail: sanotomo@nies.go.jp; 3 Division of Food Additives, National Institute of Health Sciences, 1-18-1 Kamiyoga, Setagaya-ku, Tokyo 158-8501, Japan; E-Mail: nsugimot@nihs.go.jp; 4 Faculty of Pharmaceutical Sciences, Teikyo Heisei University, Nakano 4-21-2, Nakano-ku, Tokyo 164-8530, Japan; E-Mail: t.nishimura@thu.ac.jp

**Keywords:** microcystin, variants, cytotoxicity, primary cultured rat hepatocytes, environmental water

## Abstract

Microcystins, which are cyclic heptapeptides produced by some cyanobacterial species from algal blooms, strongly inhibit serine/threonine protein phosphatase and are known as hepatotoxins. Microcystins have many structural variations, yet insufficient information is available on the differences in the cytotoxic potentials among the structural variants. In this study, the cytotoxicities of 16 microcystin variants at concentrations of 0.03–10 μg/mL to primary cultured rat hepatocytes were determined by measuring cellular ATP content, and subsequently determined by their 50% inhibitory concentration (IC_50_). Differences in the amino acid constituents were associated with differences in cytotoxic potential. [d-Asp^3^, *Z*-Dhb^7^] microcystin-LR exhibited the strongest cytotoxicity at IC_50_ of 0.053 μg/mL among the microcystin variants tested. Furthermore, [d-Asp^3^, *Z*-Dhb^7^] microcystin-HtyR was also highly cytotoxic. These results suggest that both d-Asp and *Z*-Dhb residues are important in determining the cytotoxic potential of microcystin variants.

## 1. Introduction

Algal blooms consist of the overgrowth of cyanobacteria in nutrient-rich standing water. When the occurring species produces toxins, these water blooms are hazardous to wildlife, livestock, humans and environmental water systems. Some cyanobacteria species, such as Microcystis, produce microcystins (MCs), which are potent hepatotoxins [1,2,3,4]. Moreover, because global warming causes an increase in the occurrence of blooms, there are concerns that MC variants may result in enhanced health hazards for people in countries under the subtropic and temperate region [5,6].

MCs are macrocyclic heptapeptides with the principal amino acids sequence, cyclo-(d-Ala^1^-l-X^2^-d-MeAsp^3^-l-Z^4^-Adda^5^-d-Glu^6^-MDha^7^), where d-MeAsp is d-erythro-β-methylaspartic acid, and Mdha is *N*-methyldehydroalanine. As an example, the sequence of MC-LR has X = leucine and Z = arginine [7,8,9]. There are many sequence variations depending on the identity of the l-amino acid at the X^2^ and Z^4^ positions as well as demethylation of d-MeAsp and/or Mdha, and these sequence variations are regarded as MC variants or congeners [10,11]. (2*S*,3*S*,8*S*,9*S*)-3-Amino-9-methoxy-2,6,8-trimethyl-10-phenyldeca-4,6-dienoic acid (Adda) is a common amino acid among the MC variants and is essential for the inhibition of protein phosphatases [12,13,14]. MCs are taken up by the specific bile acid transport system to hepatocytes [15,16]. Binding selectively to protein phosphatase 1 and 2A and inhibiting phosphatase activity, as a result, MCs cause severe damage to the liver [17,18]. MCs have also been reported to promote the development of liver tumors [17,19]. Moreover, they were reported that MCs have genotoxic potentials [20,21,22] and affects other organs such as heart, kidney and lung [21,23,24].

MC-LR is one of the first detected microcystins, and many studies on its toxic effects have since been conducted both *in vitro* [20,25,26] and *in vivo* [18,27,28,29,30]. The World Health Organization (WHO) adopted a provisional guideline value of 1 μg/L for a maximum concentration of MC-LR in drinking water [31]. A couple of reports evaluated the toxicity of each MC variant as a relative value to that of the toxicity of MC-LR [10,32]. Numerous MC variants have been identified from environmental water around the world [33,34,35,36]. Therefore it is important to evaluate the toxicity and environmental behavior of MCs and variants in environment water. However, there is not sufficient information of the cytotoxicity in various MC variants, although some studies for cytotoxicities of MC variants were reported [37,38,39].

In this study, we evaluated the cytotoxicity of 16 MC variants to primary cultured rat hepatocytes. Primary cultured rat hepatocytes were chosen in this study because there were reported that MC-LR induces severe toxicity to the rat liver *in vivo* [28,29] and causes cytotoxic effects in the hepatocyte [22,40,41]. Moreover, in the present study, we examined the correlation between the cytotoxicity and the amino acid constituents of the MC variants.

## 2. Results

**Figure 1:**
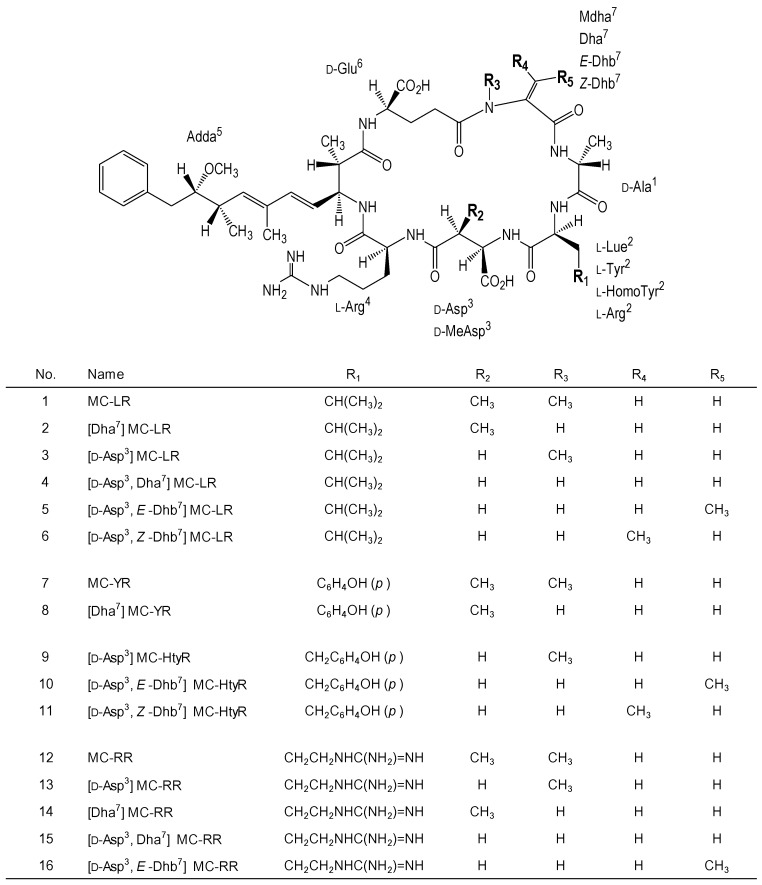
Structures of the MC variants. MC variants are cyclic peptides consisting of seven amino acids. The sequence position numbers of the amino acids number are denoted by a superscript.

**Figure 2:**
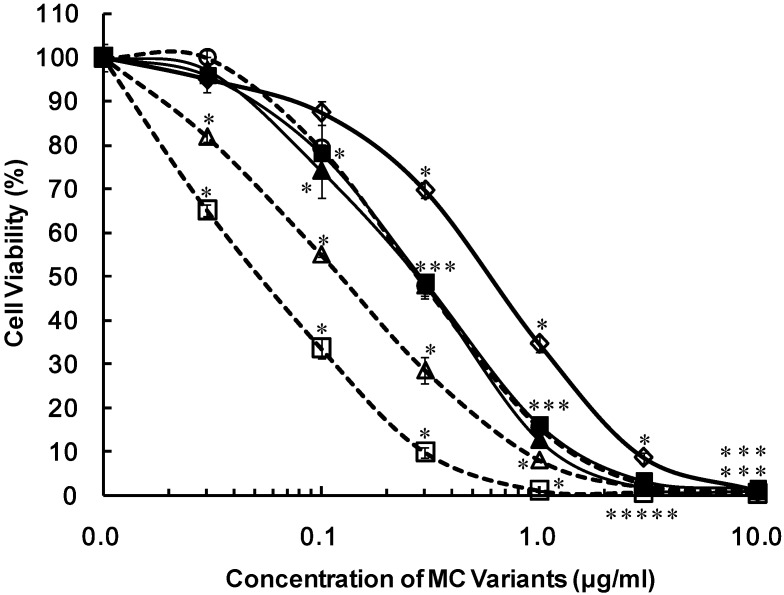
Cytotoxicities of the MC-LR variants in primary cultured rat hepatocytes after 72-h exposure, determined by the cell viability assay. Primary cultured rat hepatocytes were exposed to purified MC-LR, [Dha^7^] MC-LR, [d-Asp^3^] MC-LR, [d-Asp^3^, Dha^7^] MC-LR, [d-Asp^3^, *E*-Dhb^7^] MC-LR, [d-Asp^3^, *Z*-Dhb^7^] MC-LR. After 72 h of exposures, cytotoxicities were determined by the cell viability assay and the values are shown as % viability. Results are presented as the mean ± SD of three independent experiments. * Significantly different from the control: *p* < 0.05. (Compounds No.1: MC-LR (◊); No.2: [Dha^7^] MC-LR (▲); No.3: [d-Asp^3^] MC-LR (○); No.4: [d-Asp^3^, Dha^7^] MC-LR (■); No.5: [d-Asp^3^, *E*-Dhb^7^] MC-LR (∆); No.6: [d-Asp^3^, *Z*-Dhb^7^] MC-LR (□)).

We evaluated the cytotoxicities of 16 MC variants. Their structures are shown in Figure 1. Figure 2 shows the dose response curves of primary cultured rat hepatocytes after exposure to five MC-LR variants, namely [Dha^7^] MC-LR, [d-Asp^3^] MC-LR, [d-Asp^3^, Dha^7^] MC-LR, [d-Asp^3^, *E*-Dhb^7^] MC-LR and [d-Asp^3^, *Z*-Dhb^7^] MC-LR. These five MC-LR variants exhibited higher cytotoxic activities than MC-LR. The IC_50_ values of the MC variants are listed in Table 1. Based on the IC_50_ values, the cytotoxic potentials of the MC-LR variants were determined. The cytotoxic potentials of the MC-LR variants are ranked as follows: [d-Asp^3^, *Z*-Dhb^7^] MC-LR > [d-Asp^3^, *E*-Dhb^7^] MC-LR > [d-Asp^3^, Dha^7^] MC-LR = [d-Asp^3^] MC-LR = [Dha^7^] MC-LR > MC-LR (Table 1).

[Dha^7^] MC-LR and [d-Asp^3^] MC-LR are demethylated at the R_3_ and R_2_ positions, respectively, compared to MC-LR, and their cytotoxic activities were found to be higher than that of MC-LR. [d-Asp^3^, *Z*-Dhb^7^] MC-LR and [d-Asp^3^, *E*-Dhb^7^] MC-LR, which contain substituted methyl groups at the R_4_ and R_5_ positions, respectively, were also highly cytotoxic (Figure 2).

**Figure 3:**
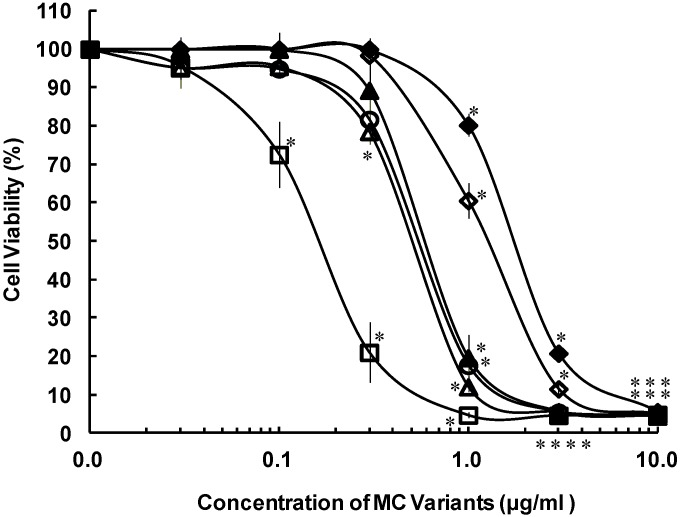
Cytotoxicities of the MC-YR variants in primary cultured rat hepatocytes after 72-h exposure, determined by the cell viability assay. Primary cultured rat hepatocytes were exposed to MC-LR, MC-YR, [Dha^7^] MC-YR, [d-Asp^3^] MC-HtyR, [d-Asp^3^, *E*-Dhb^7^] MC-HtyR, [d-Asp^3^, *Z*-Dhb^7^] MC-YR. After 72 h of exposures, cytotoxicities were determined by the cell viability assay and the values were shown as % viability. Results are presented as the mean ± SD of three independent experiments. * Significantly different from the control: *p* < 0.05. (Compounds No.1: MC-LR (◊); No.7: MC-YR (♦); No.8: [Dha^7^] MC-YR (▲); No.9: [d-Asp^3^] MC-HtyR (○); No.10: [d-Asp^3^, *E*-Dhb^7^] MC-HtyR (∆); No.11: [D-Asp^3^, *Z*-Dhb^7^] MC-HtyR (□)).

[Dha^7^] MC-YR and three MC-HtyR variants showed higher cytotoxic activities than those of MC-YR and -LR (Figure 3). The IC_50_ values are ranked as follows: MC-YR > [Dha^7^] MC-YR > [d-Asp^3^] MC-HtyR > [d-Asp^3^, *E*-Dhb^7^] MC-HtyR > [d-Asp^3^, *Z*-Dhb^7^] MC-HtyR (Table 1). [Dha^7^] MC-YR, which is a demethylated variant (at the R_3_ position) of MC-YR, caused an increase in cytotoxic activity relative to MC-YR (Figure 1 and Figure 3).

[d-Asp^3^, *Z*-Dhb^7^] MC-HtyR, which has a methyl group at the R_4_ position, exhibited significantly high cytotoxic activity, compared to [d-Asp^3^, *E*-Dhb^7^] MC-HtyR, which has a methyl group at the R_5_ position (Figure 1 and Figure 3).

**Figure 4:**
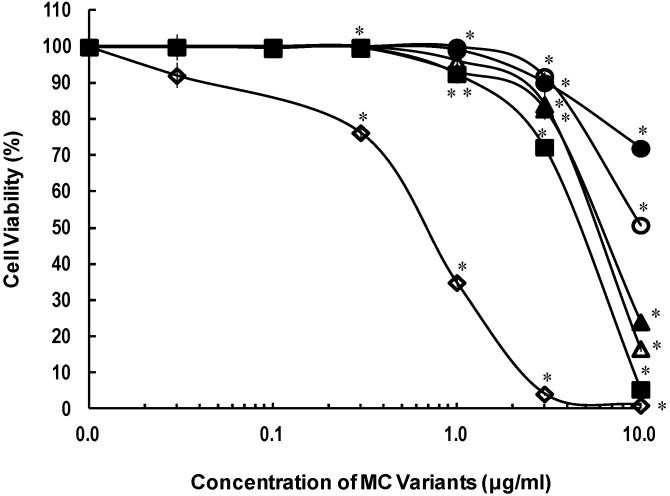
Cytotoxicities of the MC-RR variants in primary cultured rat hepatocytes after 72-h exposure, determined by the cell viability assay. Primary cultured rat hepatocytes were exposed to MC-LR, MC-RR, [d-Asp^3^] MC-RR, [Dha^7^] MC-RR, [d-Asp^3^, Dha^7^] MC-RR, [d-Asp^3^, *E*-Dhb^7^] MC-RR. After 72 h of exposures, cytotoxicities were determined by the cell viability assay and the values were shown as % viability. Results are presented as the mean ± SD of three independent experiments. * Significantly different from the control: *p* < 0.05. (Compounds No.1: MC-LR (◊); No.12: MC-RR (●); No.13: [d-Asp^3^] MC-RR (○); No.14: [Dha^7^] MC-RR (▲); No. 15: [d-Asp^3^, Dha^7^] MC-RR (■); No.16, [d-Asp^3^, *E*-Dhb^7^] MC-RR (∆)).

MC-RR was less cytotoxic than both MC-LR and -YR (Table 1). Four MC-RR variants exhibited higher cytotoxic activities than MC-RR (Figure 4). [Dha^7^] MC-RR, which lacks a methyl group at the R_3_ position of MC-RR, induced a slightly high cytotoxic activity relative to [d-Asp^3^] MC-RR, which lacks a methyl group at the R_2_ position of MC-RR (Figure 4).

**Table 1:** Comparison of the IC_50_ of MC variants. Each IC_50_ value was estimated from the 50% viability concentration shown in Figure 2, Figure 3 and Figure 4. Data are arranged in the order of increasing cytotoxic activity.

No.	MC variants name	IC_50_ (μg/mL)
6	[d-Asp^3^, *Z*-Dhb^7^] MC-LR	0.053
11	[d-Asp^3^, *Z*-Dhb^7^] MC-HtyR	0.120
5	[d-Asp^3^, *E*-Dhb^7^] MC-LR	0.133
4	[d-Asp^3^, Dha^7^] MC-LR	0.217
3	[d-Asp^3^] MC-LR	0.217
2	[Dha^7^] MC-LR	0.217
10	[d-Asp^3^, *E*-Dhb^7^] MC-HtyR	0.327
9	[d-Asp^3^] MC-HtyR	0.347
8	[dha^7^] MC-YR	0.418
1	MC-LR	0.800
7	MC-YR	1.48
15	[d-Asp^3^, Dha^7^] MC-RR	4.11
16	[d-Asp^3^, *E*-Dhb^7^] MC-RR	4.95
14	[Dha^7^] MC-RR	5.33
13	[d-Asp^3^] MC-RR	>10
12	MC-RR	>10

## 3. Discussion

We evaluated the cytotoxicities of MC variants isolated from some cyanobacteria to primary cultured rat hepatocytes. Accumulation of toxicity information for MC variants is absolutely essential to conduct the risk assessment or management of them. Therefore, toxic properties of 16 MV variants indicated in this study would be helpful basic information for the risk assessment or management of many MC variants in environmental water.

In this study, IC_50_ of MC-LR to primary rat hepatocytes was 0.8 μg/mL, however earlier study was reported that IC_50_ of MC-LR to the same cells was 48 ng/mL [42]. The MTT assay used in the earlier study and the assay which measure the cellular ATP contents (ATP assay) show occasional different IC_50_ with certain differences of cell lines, exposure chemicals, number of cells *etc.* [43,44]. Therefore, the difference of IC_50_ might be attributed to these assay methods to evaluate the cell viability. Moreover, in this study, we used the freeze-thawed cells and seeded 2.5-fold of cell number compared to the cell number in the earlier study. These differences might have caused the reduction of the cytotoxicity. We think that it is important to evaluate the result taking the difference of the experimental methods into consideration in the risk assessment or management.

Lack of a methyl group at either the R_3_ or R_2_ position of Dha^7^ or d-Asp^3^, resulted in enhanced cytotoxic activities of MC-LR, -YR, and -RR. In particular, for MC-RR, the lack of a methyl group at the R_3_ position might be responsible for the slight enhancement of cytotoxic activity, compared to demethylation at the R_2_ position (Figure 4).

It has been reported that [Asp^3^, Dhb^7^] MC-RR exhibits higher cytotoxicity than MC-RR [45,46]. In this study, d-Asp^3^ and *E*-Dhb^7^ or d-Asp^3^ and *Z*-Dhb^7^ on MC-LR and -HtyR also induced higher cytotoxic activities than d-Asp^3^ or Dha^7^ on MC-LR and -HtyR. These results suggest that substitution of the methyl group at the R_4_ or R_5_ positions is associated with cytotoxic potential, rather than the lack of a methyl group at the R_3_ or R_2_ positions. In fact, [d-Asp^3^, *Z*-Dhb^7^] MC-LR exhibited the strongest cytotoxic activity among the 16 variants, and [d-Asp^3^, *Z*-Dhb^7^] MC-HtyR also exhibited high cytotoxicity. A comparison of the IC_50_ values among [d-Asp^3^, *Z*-Dhb^7^] and [d-Asp^3^, *E*-Dhb^7^] on MC-LR and -HtyR suggests that the presence of a methyl group at the R_4_ position caused stronger effects on cytotoxic activity than the presence of a methyl group at the R_5_ position (Table 1). Although the d-Asp^3^ residue may be essential for the enhancement of cytotoxicity, the R_4_ methyl group of the *Z*-Dhb^7^ residue may also play a key role in the cytotoxic activities of MCs such as the Adda moiety.

Distinct properties of MC variants in terms of uptake, cytotoxicity, protein phosphatase 1 and 2A inhibition, were reported in other organisms cells, brain and neuron cells of mice or Caco-2 cells from human [37,38,39,47]. It is thought that MC variants with the same hydrophobic properties show the same permeabilities to the cell membrane. Vesterkvist *et al.* suggested that the hydrophobic MC has pronounced cytotoxic potentials in Caco-2 cells [38]. Since [d-Asp^3^, *Z*-Dhb^7^]-MC-LR and -HtyR are geometric isomers of [d-Asp^3^, *E*-Dhb^7^]-MC-LR and -HtyR, respectively, they have same hydrophobic property. However, the *Z* geometric isomers had more potent cytotoxic activities than the *E* geometric isomers in primary cultured rat hepatocytes in our study. Therefore, at R_4_ and R_5_ position of 7th amino acid (Mdha, Dha or Dhb), the conformational property would be an important factor for cytotoxicity to rat hepatocytes. Differences in the response of the proteins on cell surface or inside the cell, which are caused by differences of the conformational property, might determine the toxic potentials. While the protein phosphatase inhibition activities of MCs were reported in various studies, it was reported that the activity with MC variants did not correlate with their toxic potential [38,46]. There is little information of cellular uptake of MC variants in each organ. More information on membrane permeability, cellular uptake in various organs or species, interaction with target proteins *etc.* is needed to fully understand different toxic potentials of MC variants.

## 4. Experimental Section

### 4.1. MC Variants

The 16 MC variants were separated and purified by high performance liquid chromatography. The details of the separation procedure have been described in a previous report [48]. MC-LR, [d-Asp^3^, Dha^7^] MC-LR, [Dha^7^] MC-LR, MC-YR, [Dha^7^] MC-YR, MC-RR, [Dha^7^] MC-RR and [d-Asp^3^, Dha^7^] MC-RR were obtained from cultured *Microcystis aeruginosa* (NIES-90). [d-Asp^3^] MC-LR, [d-Asp^3^] MC-HtyR and [d-Asp^3^] MC-RR were obtained from *Planktothrix agardhii* (NIES-595 and NIES-1263). [d-Asp^3^, *Z*-Dhb^7^] MC-LR and [d-Asp^3^, *Z*-Dhb^7^] MC-HtyR were isolated from bloom of *Planktothrix agardhii* [49]. [d-Asp^3^, *E*-Dhb^7^] MC-LR, [d-Asp^3^, *E*-Dhb^7^] MC-HtyR and [d-Asp^3^, *E*-Dhb^7^] MC-RR were obtained from cultured *Planktothrix rubescens* (NIES-610 and NIES-928). The accurate concentrations of isolated MC variant solutions were determined based on the absorbance at 238 nm using the molar extinction coefficient (42000). The structures of these MC variants are shown in Figure 1.

### 4.2. Cells

Primary cultured rat hepatocytes, which maintain both phase I and II metabolic activity as well as uptake transporter activity, were isolated from *Sprague Dawley*. The cells were purchased from Biopredic International (Rennes, France) and were stored at −135 °C until further use. Thawing medium, seeding medium, and long-term culture medium were purchased from Biopredic International (Rennes, France). The primary cultured rat hepatocytes were thawed in a water bath at 37 °C. The cells were poured into 30 mL of pre-warmed thawing medium. The cell suspension was centrifuged at 900 rpm (160 × *g*) for 1 min. The supernatant was removed and the cell pellets were re-suspended in 2 mL of the seeding medium. The cells were seeded in 96-well white plates at 0.3 × 10^6^ cells/mL in 0.1 mL/well of the seeding medium and incubated at 37 °C in 5% CO_2_. The following day, the cells were exposed to sixteen MC variants with the long-term culture medium.

### 4.3. Cytotoxicity Assay

MC variants were dissolved in a mixture of DMSO:water = 9:1 (*v*/*v*) to make 1.0 mg/mL stock solutions taking the solubility of MC variants to DMSO into consideration. Stock solutions were diluted to 3–1000 μg/mL with DMSO, and subsequently diluted to final concentrations of 0.03–10 μg/mL in the medium. 1% DMSO solution was diluted 100-fold with the medium, and the resulting solution was used as a negative control. Cells were seeded in 96-well white plates and pre-cultured at 37 °C in 5% CO_2_ overnight. Cells were then exposed to various concentrations of MC variants in the long-term culture medium for 72 h. Cytotoxicity was evaluated using the Cell Titer-Glo Luminescent Cell Viability Assay Kit (Promega Corporation, Madison, WI, USA), based on cellular ATP content. After 10 min of incubation with an equal volume of the Cell Titer-Glo reagent at room temperature, luminescence was measured for 1 s/well. The luminescence intensity of each well treated with an MC variant was determined, relative to the control, and those concentrations of the MC variants that showed 50% cell viability (IC_50_) were calculated. MC-LR was used as a positive reference substance for every assay.

### 4.4. Statistical Analysis

All experiments values were expressed as the mean ± standard deviations (SD) of three independent experiments. The results were analyzed statistically with the Student’s *t*-test. The test was conducted to verify the difference between each group exposed to MC variants and the control. Differences with *p* < 0.05 were considered statistical significant.

## 5. Conclusions

We have determined the cytotoxic potentials of 16 types of MC variants primary culture rat hepatocytes. MC variants containing d-Asp^3^ and Dha^7^ and/or Dhb^7^ residues were found to exhibit stronger cytotoxic activities than their corresponding normal MC variants. The *Z*-Dhb^7^ residue of the MC variants is important for their cytotoxic potential. Toxic properties of 16 MC variants indicated in this study would help accumulation of toxicity basic information on various MCs, and eventually contribute to the risk assessment and/or management of environmental water.
